# Prevalence of respiratory viruses and antiviral MxA responses in children with febrile urinary tract infection

**DOI:** 10.1007/s10096-020-03836-5

**Published:** 2020-02-11

**Authors:** Ruut Piri, Lauri Ivaska, Mohamed Yahya, Laura Toivonen, Johanna Lempainen, Janne Kataja, Kirsi Nuolivirta, Lav Tripathi, Matti Waris, Ville Peltola

**Affiliations:** 1 Department of Paediatrics and Adolescent Medicine, Turku University Hospital and University of Turku, 20521 Turku, Finland; 2 Institute of Biomedicine, University of Turku and Clinical Microbiology, Turku University Hospital, Turku, Finland; 3 Department of Paediatrics, Seinäjoki Central Hospital, Seinäjoki, Finland; 4 Institute of Biomedicine, University of Turku, Turku, Finland

**Keywords:** Infant, Interferon-inducible protein, Myxovirus resistance protein A, Pyelonephritis, Respiratory tract infection

## Abstract

Blood myxovirus resistance protein A (MxA) has broad antiviral activity, and it is a potential biomarker for symptomatic virus infections. Limited data is available of MxA in coinciding viral and bacterial infections. We investigated blood MxA levels in children hospitalized with a febrile urinary tract infection (UTI) with or without simultaneous respiratory virus infection. We conducted a prospective observational study of 43 children hospitalized with febrile UTI. Nasopharyngeal swab samples were collected at admission and tested for 16 respiratory viruses by nucleic acid detection methods. Respiratory symptoms were recorded, and blood MxA levels were determined. The median age of study children was 4 months (interquartile range, 2–14 months). A respiratory virus was detected in 17 (40%) children with febrile UTI. Of the virus-positive children with febrile UTI, 7 (41%) had simultaneous respiratory symptoms. Blood MxA levels were higher in virus-positive children with respiratory symptoms (median, 778 [interquartile range, 535–2538] μg/L) compared to either virus-negative (155 [94–301] μg/L, *P* < 0.001) or virus-positive (171 [112–331] μg/L, *P* = 0.006) children without respiratory symptoms at presentation with febrile UTI. MxA differentiated virus-positive children with respiratory symptoms from virus-negative without symptoms by an area under the receiver operating characteristic curve of 0.96. Respiratory viruses were frequently detected in children with febrile UTI. In UTI with simultaneous respiratory symptoms, host antiviral immune response was demonstrated by elevated blood MxA protein levels. MxA protein could be a robust biomarker of symptomatic viral infection in children with febrile UTI.

## Introduction

Febrile urinary tract infection (UTI) is the most common serious bacterial infection in children [1–3]. Febrile UTI can occur simultaneously with a respiratory tract infection, but the likelihood of UTI has been reported to be significantly lower among respiratory virus-positive than in virus-negative febrile infants [4, 5]. Highly sensitive multiplex polymerase chain reaction (PCR) assays and other nucleic acid amplification tests with short turnaround times are currently widely used in hospitals and emergency departments (EDs) for detecting respiratory viruses. However, positive results must be interpreted with caution since certain respiratory viruses, like rhinoviruses, coronaviruses, and human bocavirus, are frequently detected also in asymptomatic children [6]. In addition to rapid and sensitive virus detection methods, a biomarker of host response to respiratory virus infection could be useful in the evaluation of febrile infants.

Myxovirus resistance protein A (MxA) is an interferon-inducible protein with antiviral activity against a wide range of viruses that cause respiratory, gastrointestinal, and generalized infections [7, 8]. It has been studied as a potential biomarker of symptomatic viral infections due to its broad antiviral range, rapid induction in 1 to 2 h after onset of symptoms, and low basal levels in healthy children [9, 10]. Blood MxA protein levels are markedly elevated in symptomatic viral infections but not in respiratory virus-positive asymptomatic children [7]. MxA response has not been extensively studied in very young infants or in children with coinciding viral and bacterial infections. In a study of acute pharyngitis, elevated blood MxA levels were found in children coinfected with a respiratory virus and group A streptococcus but not in children with only group A streptococcus infection [11].

The aim of this study was to investigate the prevalence of symptomatic and asymptomatic respiratory virus infections in children hospitalized with febrile UTI and to estimate antiviral immune responses by the blood MxA protein levels.

## Materials and methods

### Study population and conduct

This study was a part of a prospective observational two-center study of biomarkers to differentiate between viral and bacterial infections in children. Children hospitalized for an acute severe infection (based on an order for blood bacterial culture) were enrolled at the pediatric EDs of Turku University Hospital and Seinäjoki Central Hospital, Finland, between December 2016 and April 2018. The inclusion criteria were (1) age between 4 weeks and 16 years, (2) admission to hospital, and (3) blood bacterial culture drawn by the decision of the attending clinician. An exclusion criterion was cancer under active treatment. For the current analysis, we included study children with a diagnosis of febrile UTI. Data on symptoms and recently administered vaccines were collected by parent-filled structured questionnaires and from the electronic registries of well-baby clinics. Documentation of respiratory symptoms (rhinorrhea, cough) was based on parental questionnaire and on clinical examination at the ED. Nasopharyngeal, blood, and urine samples were collected at the ED.

The study protocol was approved by the Ethics Committee of the Hospital District of Southwest Finland. The parents of all children and children or adolescents themselves, if old enough, provided their written informed consent at the enrolment.

### Diagnosis of UTI

Urine samples were collected either by a suprapubic aspiration, a urine collection bag, or as a clean voided midstream specimen and analyzed by standard flow cytometry and culture methods at the hospital laboratories. A definite febrile UTI was defined as fever and a positive culture of urine obtained by suprapubic aspiration or as fever, pyuria, and a positive culture of urine obtained by a collection bag or as a midstream specimen. A positive urine culture was defined as any bacterial growth in a suprapubic aspirate or as a growth of ≥ 10^4^ colony-forming units per mL of a single uropathogen species in a sample obtained by a collection bag or as a midstream specimen. Pyuria was defined as a positive leucocyte esterase test or as a count of ≥ 30 × 10^6^ white blood cells (WBC)/L urine (approximately equivalent to 5 WBCs per high-power field in microscopic analysis). A probable febrile UTI was defined as fever and a positive urine culture but no pyuria in a sample obtained by a collection bag or as a midstream specimen.

### Virus detection

Nasopharyngeal swab samples were suspended into phosphate-buffered saline, and nucleic acids were extracted using NucliSENS easyMag (bioMerieux, Boxtel, Netherlands). Allplex respiratory panel multiplex PCR (Seegene, Seoul, South Korea) was used in the Turku University Hospital and FilmArray (BioFire Diagnostics, Salt Lake City, UT) in Seinäjoki Central Hospital for the detection of respiratory viruses. Both methods detected adenovirus; influenza A and B viruses; parainfluenza viruses type 1, 2, 3, and 4; respiratory syncytial virus; human metapneumovirus, coronaviruses V2293, NL62, and OC43; rhinovirus; and enteroviruses. Allplex also detected human bocavirus and Filmarray coronavirus HKU1. As FilmArray detected picornaviruses together, samples positive for rhinovirus/enterovirus by this method were further analyzed with Allplex to make a specific diagnosis of either rhinovirus or enterovirus.

### Biomarker measurements

Blood samples for bacterial culture, WBC count, plasma C-reactive protein (CRP), plasma procalcitonin (PCT), and blood MxA protein levels were collected by venous puncture. Blood bacterial culture, WBC count, and plasma CRP and PCT levels were determined by routine methods in the hospital central laboratory. Whole blood samples for MxA protein measurement were diluted 1:20 in hypotonic buffer and stored at − 70 °C until the enzyme immunoassay analysis was performed as described earlier [7]. Samples with a level exceeding the range of the assay in primary analysis were retested at a 1:200 dilution.

### Statistical analyses

Blood MxA protein levels were compared in children with febrile UTIs grouped by respiratory symptoms and virus findings using Kruskal-Wallis test followed by pairwise Mann-Whitney U test. *P* values were adjusted for multiple comparisons with the use of Bonferroni correction. Receiver operating characteristic (ROC) analysis was used to evaluate the capability of blood MxA protein level to distinguish virus-positive children with respiratory symptoms from virus-negative children without respiratory symptoms and to discriminate between virus-positive children with or without respiratory symptoms. A cutoff level for blood MxA protein was calculated from the ROC analysis using Youden index (sensitivity + specificity – 1). Two-tailed *P* values < 0.05 were considered statistically significant. Statistical analyses were performed using IBM SPSS Statistics, version 25.0 (IBM Corp., Armonk, NY).

## Results

### Clinical and microbiological characteristics

In the main study, 273 children were recruited, and 48 of them were diagnosed with febrile UTI. Four children were excluded because of a missing nasopharyngeal swab sample and one child for a missing blood MxA sample. Of the 43 children included in the analysis, 42 (98%) were classified as having a definite UTI and one (2%) as having a probable UTI (Table 1).

**Table 1:** Characteristics and biomarker levels of children with febrile UTI

Variable	Study children (*n* = 43)
Age group (months), *n* (%)	
< 3	14 (33)
3–11	17 (40)
12–35	7 (16)
≥ 36	5 (12)
Sex, female, *n* (%)	23 (54)
History of febrile UTI, *n* (%)	7 (16)
Anomalies of the kidney or urinary tract, *n* (%)	10 (23)
Live attenuated vaccine in past 30 days, *n* (%)*	19 (44)
Urosepsis, *n* (%)	2 (4)
Symptoms of respiratory tract infection, *n* (%)	11 (26)
Duration of fever before admission (days), median (IQR)	1.0 (0–3.0)
Highest measured body temperature (°C), median (IQR)	39.4 (38.8–40.0)
Respiratory virus detected, *n* (%)	17 (40)
CRP upon admission (mg/L), median (IQR)	81 (30–122)
PCT upon admission (μg/L), median (IQR)	0.7 (0.2–4.7)
MxA upon admission (μg/L), median (IQR)	253 (116–458)

*All children recently immunized with a live vaccine had received an oral rotavirus vaccine

The median age of study children was 4 months (interquartile range [IQR], 2–14 months). The median [IQR] ages in children grouped by respiratory symptoms and virus findings were as follows: virus-negative children without respiratory symptoms, 4 [2–20] months; virus-positive children without respiratory symptoms, 8 [2–28] months; virus-positive children with respiratory symptoms, 3 [3–5] months; and virus-negative children with respiratory symptoms, 3 [2–11] months (*P* = 0.73). The most common pathogen detected in urine cultures was *Escherichia coli* (*n* = 33) followed by *Enterococcus* species (*n* = 4) and *Klebsiella pneumoniae* (*n* = 2). A respiratory virus was detected in nasopharyngeal samples of 17 (40%) children with a febrile UTI. Rhinovirus was detected in 11 children, coronavirus NL63 or OC43 in 4, human bocavirus in 2, respiratory syncytial virus in 2, influenza B virus in 1, adenovirus in 1, and parainfluenza virus type 1 in 1 child. Multiple viruses were detected in 4 (9%) children. Symptoms of a respiratory tract infection were present in 11 (26%) children with a febrile UTI, in 7 (41%) of 17 virus-positive children, and in 4 (15%) of 26 children without a detected virus. At least one respiratory virus was detected in 64% of children with respiratory symptoms and in 31% of children without respiratory symptoms.

### Blood MxA protein as a marker of virus infection

**Fig. 1:**
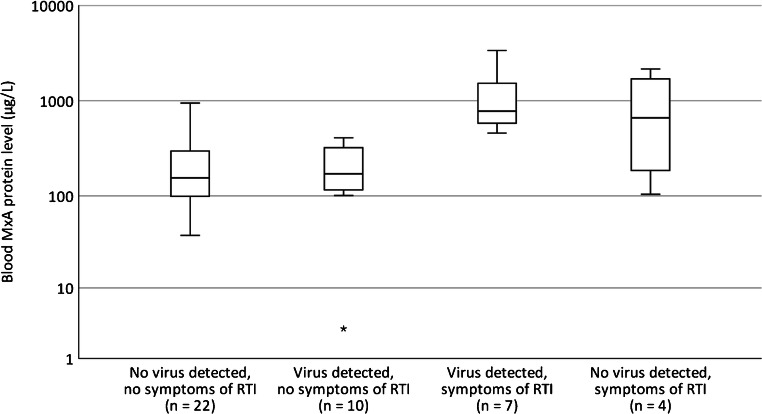
Boxplot graph (median, IQR, 95% CI, outliers) of blood MxA protein levels in 43 children with febrile UTI according to the detection of viruses and the presence of symptoms of respiratory tract infection (RTI). Differences between groups were significant by Kruskal-Wallis test. For pairwise comparisons of group “Virus detected, symptoms of RTI” with “No virus detected, no symptoms of RTI”, *P* < 0.001, and with “Virus detected, no symptoms of RTI”, *P =* 0.006 by Mann-Whitney U test

The blood MxA protein levels in children with febrile UTI according to the detection of viruses and the presence of respiratory symptoms are shown in Fig. 1. Blood MxA levels (median [IQR]) were significantly higher in virus-positive children with respiratory symptoms (778 [535–2538] μg/L) compared to either virus-negative (155 [94–301] μg/L, *P* < 0.001) or virus-positive (171 [112–331] μg/L, *P* = 0.006) children without respiratory symptoms. There was no significant difference in the blood MxA levels in virus-negative vs. virus-positive children without respiratory symptoms (*P* = 1.0). Seven (16%) of 43 study children had a symptomatic, PCR-positive viral infection with an elevated blood MxA protein level (range 458–3367 μg/L) simultaneously with a UTI.

**Fig. 2:**
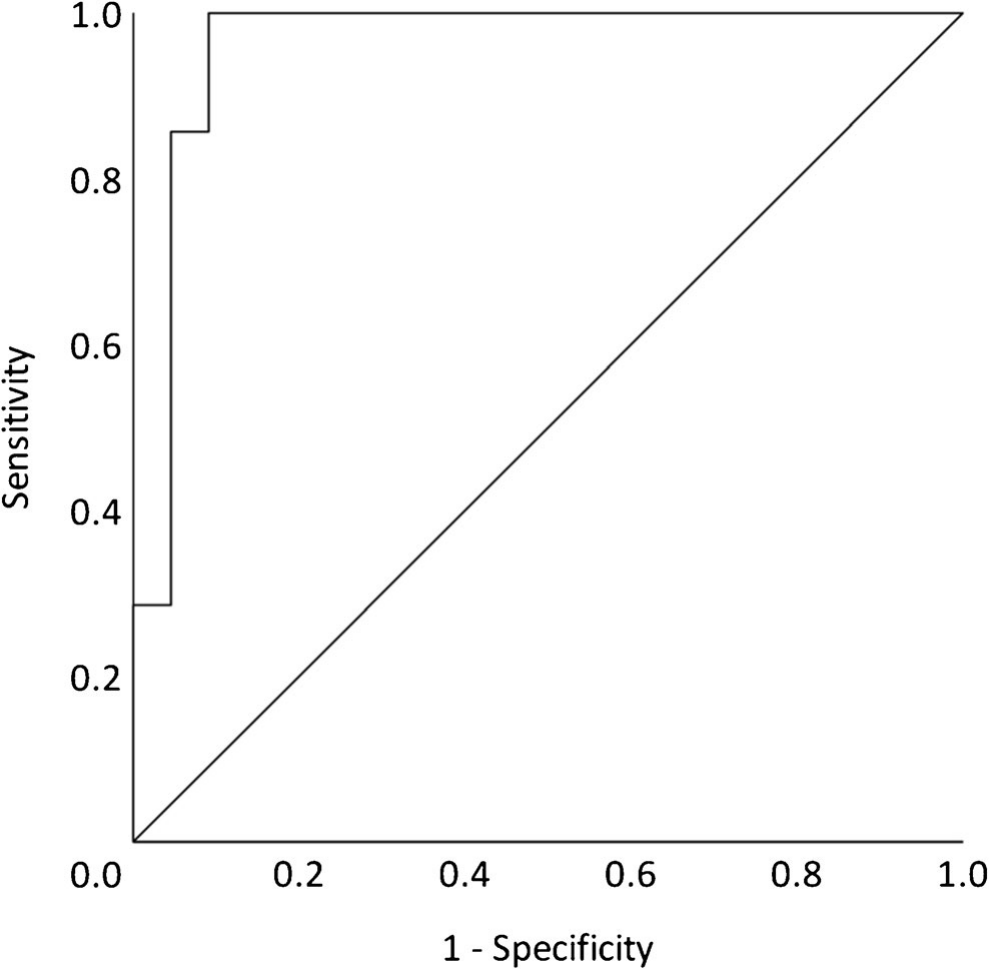
ROC analysis showing the ability of blood MxA protein level to differentiate virus-positive children with febrile UTI and respiratory symptoms from virus-negative children with febrile UTI and without respiratory symptoms. Area under curve (AUC), 0.96 (95% CI, 0.89–1.0)

ROC analysis was done to estimate the ability of blood MxA protein to differentiate virus-positive children with respiratory symptoms from virus-negative children without respiratory symptoms. The area under the ROC curve (AUC) was 0.96 (95% confidence interval [CI], 0.89–1.0), and the greatest sum of sensitivity (100%) and specificity (90.9%) was obtained with a cutoff level of 409 μg/L (Fig. 2). With a cutoff level of 220 μg/L, which is close to the cutoff of 200 μg/L set by Engelman et al. in their study comparing children with viral or bacterial infections [8], the sensitivity was 100%, and the specificity was 59%. When discriminating between virus-positive children with or without respiratory symptoms, the AUC was 1.0 (95% CI, 1.0–1.0), and a cutoff level of 434 μg/L gave sensitivity and specificity of 100%.

### Effects of live vaccinations and age on blood MxA protein

Nineteen (44%) children had received live attenuated oral rotavirus vaccine within 30 days prior to entering the study. None had received other live vaccines during this period. When children with respiratory symptoms were excluded from the analyses, there was no significant difference in the median [IQR] blood MxA level in children (*n* = 12) recently vaccinated with rotavirus vaccine (219 [140–322] μg/L) compared to children (*n* = 24) without a preceding live virus vaccination (140 [83–275] μg/L, *P* = 0.18). In children without respiratory symptoms, the median [IQR] blood MxA levels were 186 [126–322] μg/L in those below the age of 12 months (*n* = 25) and 129 [66–191] μg/L (*P* = 0.074) in those at or above the age of 12 months (*n* = 7).

## Discussion

We frequently found respiratory viruses by multiplex PCR panels in children with febrile UTI. At least one respiratory virus was detected in 40% of children hospitalized with febrile UTI; however, nearly half of the virus-positive children did not have any focal symptoms of a respiratory tract infection. Antiviral immune response of the host was demonstrated by elevated blood MxA protein level in all virus-positive children who had respiratory symptoms. Our results emphasize the high detection rate of respiratory viruses using multiplex PCR tests in nasopharyngeal samples of children with febrile UTI and show the potential of blood MxA protein as a biomarker of a symptomatic viral infection.

To our knowledge, this is the first study to examine symptoms of respiratory tract infection, respiratory viruses, and antiviral immune responses in children with febrile UTI. Earlier studies have investigated the risk of serious bacterial infection in febrile infants with or without respiratory virus infection. The presence of a respiratory virus infection has been reported to reduce the probability of UTI among febrile infants in comparison with virus-negative children [4, 5], but there are differences between viruses in this regard. The risk of concomitant UTI in infants with respiratory syncytial virus or influenza virus infection, detected either by PCR or by an antigen test, is lower than the risk in virus-negative children [12–14]. On the contrary, in another study, the probability of UTI was not substantially lower in rhinovirus-positive compared with virus-negative infants [14].

Multiplex respiratory virus PCR panels include viruses, which frequently cause asymptomatic or mildly symptomatic infections, such as rhinovirus or coronavirus, and viruses that persist in the airways, such as human bocavirus, and positive results for at least one virus are common also in healthy children [6, 7]. The perception that virus positivity reduces the risk of UTI or other serious bacterial infection possibly does not apply if febrile children are systematically tested with broad multiplex PCR panels. Our results of frequent respiratory virus findings with or without respiratory symptoms in children with UTI support this view. As expected by its frequency in all young children [15], rhinovirus was the most commonly detected virus. Our results call for diagnostic stewardship in terms of whom to test for respiratory viruses by using highly multiplexed, sensitive diagnostic assays. If multiplex PCR is routinely used in febrile children in the ED, blood MxA protein could serve as a useful biomarker for a symptomatic viral infection.

We report somewhat higher cutoff levels for blood MxA protein than previous studies [7, 8]. We examined mainly infants, but age does not seem to fully explain this finding. We did not find any large differences in MxA levels in infants below the age of 12 months compared to older children when those with respiratory symptoms were excluded. This is consistent with our previous study, in which we found higher baseline levels of blood MxA protein in children compared to adults but similar levels at ages 2 and 13 months [7]. It is important to consider the role of rotavirus or other live virus vaccinations in studies of MxA protein or other interferon-inducible proteins in children, as live vaccines can increase blood MxA levels temporarily [7, 16]. However, recent rotavirus vaccination had only minor effect on blood MxA protein levels in the present study, similarly to our earlier observations [7]. We also earlier reported that febrile respiratory infections were associated with substantially higher blood MxA levels than infections without fever [7]. The current study included febrile children only, and although the cause of fever was presumably UTI in most children, some children may have been febrile because of a simultaneous viral infection. This could have affected the blood MxA levels in children with no apparent respiratory symptoms.

Our study has certain limitations. First, despite using aseptic techniques to collect urine samples, we cannot rule out the possibility of contamination or asymptomatic bacteriuria (if symptoms were caused by a virus infection) being labeled as a UTI. Second, the relatively small number of children limits the generalizability of our results. Third, a further study with a comparison group without UTI would help in understanding whether the risk of UTI is reduced in febrile children with respiratory virus infection and elevated blood MxA level.

In conclusion, our study showed that respiratory viruses are frequently detected in children with febrile UTI when using multiplex PCR methods. An antiviral host response was demonstrated by elevated blood MxA levels in children with UTI and simultaneous respiratory symptoms. Blood MxA protein could be a robust biomarker of symptomatic viral infection in children with febrile UTI. Its use in evaluation of febrile infants deserves further study.
